# Temporal patterns of commonly used clinical outcome scales during a 5-year period after total knee arthroplasty

**DOI:** 10.1186/s10195-019-0520-8

**Published:** 2019-03-25

**Authors:** Vivek Tiwari, Jonggeun Lee, Gaurav Sharma, Yeon Gwi Kang, Tae Kyun Kim

**Affiliations:** 1 Joint Reconstruction Center, Seoul National University Bundang Hospital, 82 Gumi-ro, 173 Beon-gil, Bundang-gu, Seongnam-si, Gyeonggi-do 13620 Republic of Korea; 2 Department of Orthopaedic Surgery, Seoul National University College of Medicine, Seoul, Republic of Korea

**Keywords:** Total knee arthroplasty, Functional outcome, Pattern, Age, Prosthesis

## Abstract

**Background:**

It is not established beyond doubt whether improvements in functional outcome after total knee arthroplasty (TKA) are maintained in the long term. We therefore investigated the temporal patterns of functional outcome [using range of motion (ROM), American Knee Society (AKS) score, Western Ontario and McMaster Universities Arthritis Index (WOMAC) score, and 36-Item Short Form Health Survey (SF-36) score] over a 5-year period after uncomplicated TKA, and whether these patterns differed by implant type and patient age.

**Materials and methods:**

This prospective study evaluated 138 patients who underwent unilateral TKA with either a mobile-bearing (MB) or fixed-bearing (FB) posterior-stabilized prosthesis. An independent investigator evaluated the functional outcome at five time points: preoperatively and at 6-month, 1-year, 2-year, and 5-year follow-up. Differences in functional outcomes between adjacent time points were evaluated by mixed-effect model repeat measurement (MMRM).

**Results:**

The different functional outcome scores showed improvement till 6 months–2 years, followed by a variable decline. In patients aged ≥ 68 years with an MB implant, most of the functional outcome scores declined between 2 and 5 years after variable initial improvement till 6 months–2 years, whereas the parameters plateaued after 2 years in those aged < 68 years and in older patients with an FB implant.

**Conclusions:**

A decline in function and pain relief occurs 2 years after TKA. This decline is more evident in older patients with an MB prosthesis. Based on these findings, we believe that use of MB implants in older patients (≥ 68 years) requires further investigation.

**Level of evidence:**

Level 3.

## Introduction

Evidence-based knowledge about the temporal pattern of functional results after total knee arthroplasty (TKA) has far-reaching clinical implications. Long-term follow-up studies after TKA have shown survival rates of up to 97% at 15 and 20 years [1, 2]. Survival rates provide important information to physicians and patients; however, it is equally important to know how well the knee will function after TKA, in both the short and long term [3]. From the surgeon’s perspective, knowing the timeline of the extent of recovery and its maintenance after uneventful TKA is important to counsel patients.

Literature provides contradicting information about the temporal pattern of improvement in functional outcome after TKA. Over the last three decades, during which TKA has been successfully performed and evolved, only a handful of studies have attempted to address this issue [3–13]. Most previous studies are limited by the fact that either only the clinician-examined Knee Society Score (KSS) was used for evaluation [4, 6, 7, 9] rather than patient-reported outcomes measures (PROMs), which are of real value to the patient, or the time points at which the patients were assessed were too spaced out, thereby potentially missing the true timeline [5, 8, 12], or the number of patients available at final follow-up was too small [11], raising concerns about whether the findings are generally applicable to society. While some studies suggest that pain and function continue to improve for 4–7 years after TKA [5, 12], others suggest that these improvements are limited to 2 years after TKA, after which they begin to decline [3, 8]. Although no evidence of the superiority of either fixed-bearing (FB) or mobile-bearing (MB) types of implant has been found with respect to range of movement (ROM), pain, stiffness, or function [14], the rate of recovery may differ according to bearing type [15]. Furthermore, TKA performed at older patient age may affect the timeline of functional recovery, given the fact that such patients have a higher medical complication rate. Considering that we had prospectively collected data of a sizable number of patients at multiple time points by using various outcome scores up to midterm follow-up of 5 years, with both FB and MB types of implant, we believed that we were in a position to address these issues.

The aims of this study are: (1) to investigate the temporal patterns of functional outcomes [using ROM, American Knee Society Score [AKS], Western Ontario and McMaster Universities Arthritis Index (WOMAC) score, and 36-Item Short Form Health Survey (SF-36) score] over a 5-year period after uncomplicated TKA, and (2) to determine whether the temporal patterns of functional outcome after TKA differ by implant type and patient age.

## Patients and methods

### Study design and setting

All patients who underwent primary TKA at our tertiary care institution from November 2004 to May 2005 were prospectively evaluated for functional outcome at five time points from preoperative assessment until 5-year follow-up. This study was approved by the institutional review board of our hospital, and informed consent for use of medical information was obtained from all patients.

### Participants/study subjects

**Fig. 1:**
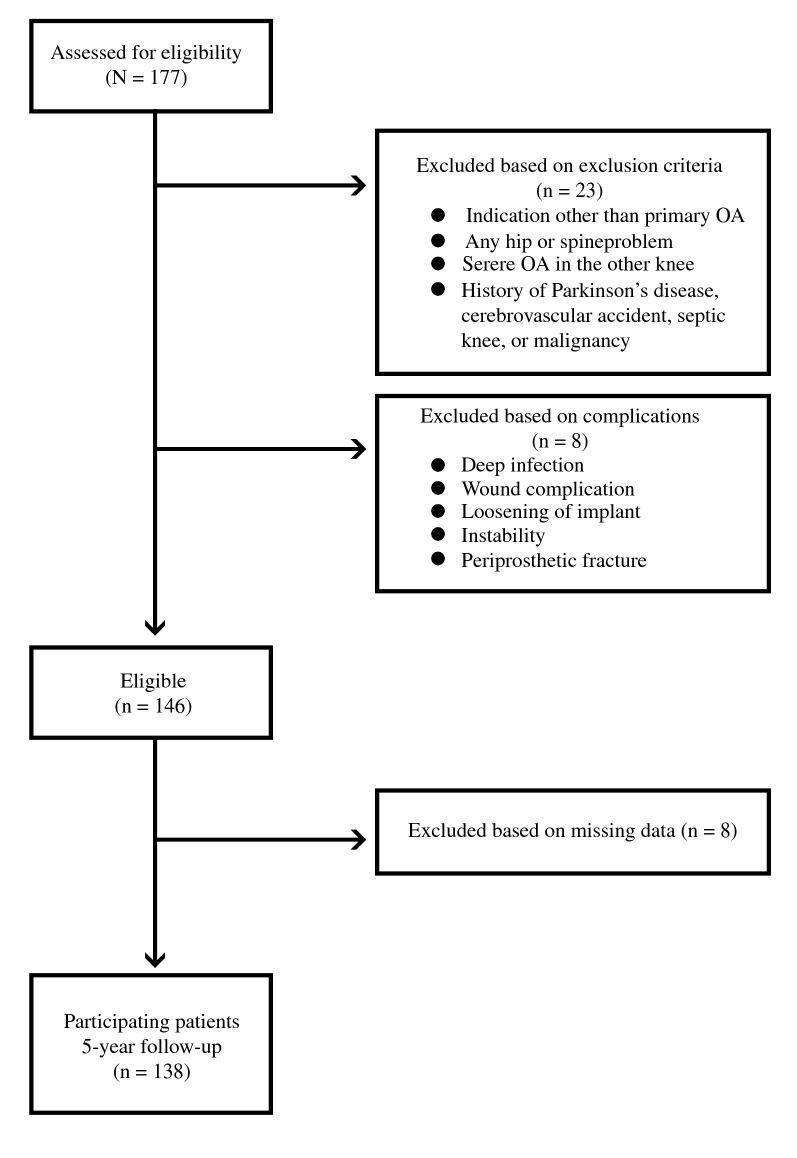
Flowchart showing details of patient enrollment and follow-up

Patients with osteoarthritis (OA) who underwent unilateral TKA during the study period and for whom outcome data were available at each of the five time points were included for analysis. During the study period, 177 patients underwent unilateral TKA at our institution and were assessed for eligibility. We excluded 23 patients on the basis of exclusion criteria and 8 patients on the basis of complications (Fig. 1). Moreover, complete follow-up data were not available for eight patients. Thus, 138 patients had complete 5-year follow-up data and were thus included in the analysis. Of the patients, 131 were female (94.9%) and 7 were male (5.1%), with mean [standard deviation (SD)] age of 67.7 (5.6) years. Their mean (SD) body weight, height, and body mass index were 62.6 (7.8) kg, 152.2 (6.0) cm, and 26.9 (3.0) kg/m^2^, respectively.

### Surgical protocol

All TKAs were performed by a single surgeon using standard medial parapatellar arthrotomy with a tourniquet. One of two posteriorly stabilized prostheses [Genesis II (FB), Smith and Nephew, Memphis, USA or e.motion (MB), B. Braun Aesculap, Tuttlingen, Germany] was implanted for each patient. Implant selection was at surgeon discretion, without any preset selection criteria. For all cases, patellar resurfacing and cement fixation were performed. A standard postoperative rehabilitation protocol was used in all patients.

### Outcome measures

Patients were evaluated by the same observer (one of the authors) at five time points, viz. preoperatively and at 6-month, 1-year, 2-year, and 5-year postoperative follow-up, by using ROM, AKS [16], WOMAC score [17], and SF-36 [18] score. The ROM of all patients was measured to the nearest 5° using a standard 38-cm clinical goniometer with the patient in supine position, calculated by subtracting the flexion contracture from maximum flexion.

### Statistical analysis

All statistical analyses were conducted by using SPSS version 21.0 for Windows (SPSS Inc., Chicago, IL); *p* value < 0.05 was considered significant. Quantitative variables are expressed as mean and SD. Differences in functional outcome between adjacent time points were evaluated by mixed-effect model repeat measurement (MMRM). To determine whether temporal patterns of functional outcomes after TKA differed by implant type and patient age, we created four groups of patients as follows: (1) patients aged < 68 years with an FB implant (*n* = 31), (2) patients aged < 68 years with an MB implant (*n* = 32), (3) patients aged ≥ 68 years with an FB implant (*n* = 38), and (4) patients aged ≥ 68 years with an MB implant (*n* = 37). The number of patients aged < 68 and ≥ 68 years was 63 and 75, respectively. Sixty-nine patients had an FB implant, while another 69 patients had an MB implant. The baseline demographic data of the two age groups and the two implant groups of patients were comparable (Tables 1 and 2). In the four groups, the functional outcome scores between adjacent time points were compared by MMRM. In comparing the functional outcomes, we considered a difference of 5° in ROM and 6% in the outcome scales to be clinically important, as the motion arc was measured to the nearest 5° and a 6% difference in maximum score has been suggested as the minimal clinically important difference for the WOMAC score and SF-36 score [19].

**Table 1:** Demographic data of patients in the two age groups

Variable	< 68 years old (*n* = 63)	≥ 68 years old (*n* = 75)	*p*-Value
Sex (female)	61 (96.8%)	70 (93.3%)	
Age (years)	62.8 (3.8)	71.7 (3.2)	< 0.001
Height (cm)	153.3 (5.4)	151.3 (6.4)	0.063
Weight (kg)	63.5 (6.7)	61.8 (8.7)	0.210
BMI (kg/m^2^)	26.9 (2.8)	27.0 (3.2)	0.911

Data presented as mean (SD) unless otherwise specified

*BMI* body mass index

**Table 2:** Demographic data of patients in the two implant groups

	MB (*n* = 69)	FB (*n* = 69)	*p*-Value
Sex (female)	64 (92.8%)	67 (97.1%)	
Age (years)	66.8 (4.8)	68.5 (6.2)	0.069
Height (cm)	152.3 (6.4)	152.1 (5.7)	0.831
Weight (kg)	62.4 (7.8)	62.7 (7.9)	0.802
BMI (kg/m^2^)	26.9 (2.9)	27.0 (3.1)	0.866

Data presented as mean (SD) unless otherwise specified

*MB* mobile bearing, *FB* fixed bearing, *BMI* body mass index

## Results

**Fig. 2:**
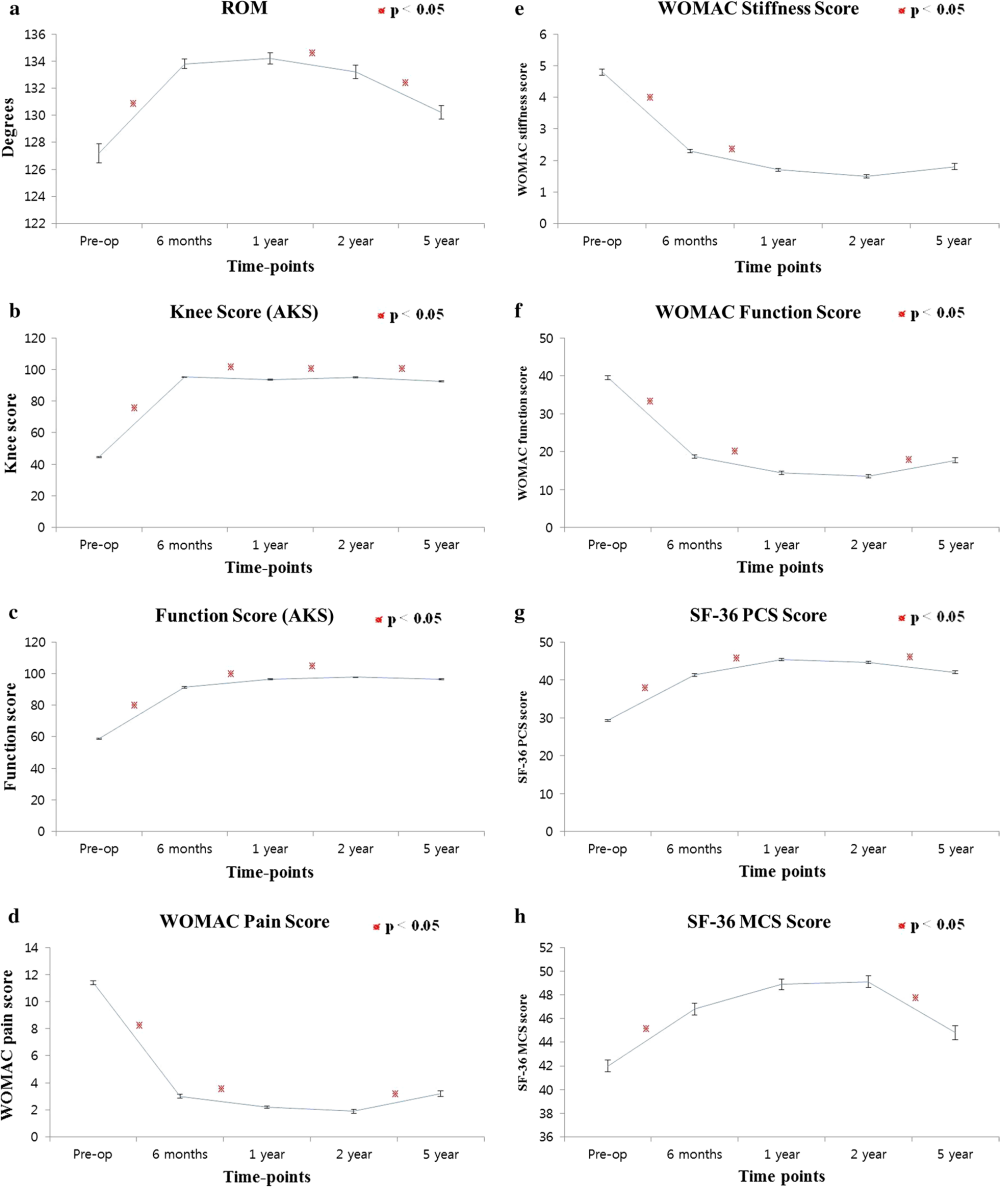
Line diagrams depicting the various outcome parameters over 5 years (error bars depict standard deviation): **a** ROM, **b** AKS knee score, **c** AKS function score; **d** WOMAC pain score, **e** WOMAC stiffness score, **f** WOMAC function score, **g** SF-36 PCS score, and **h** SF-36 MCS score

The different functional outcome scores showed improvement up to 6 months–2 years, after which most of the parameters declined (Table 3). Before operation, the mean flexion contracture was 12° with further flexion of up to 139° (ROM, 127.2°). The mean ROM showed improvement at 6 months (133.8°) and was constant after that till 1 year (134.2°), before demonstrating a decline at 2 years (133.2°) and 5 years (130.2°), although the decline was not clinically important (Fig. 2a). The mean AKS knee score showed improvement at 6 months, a small decline at 1 year, then improvement again at 2 years, reaching the 6-month level. However, it again demonstrated a decline between 2 and 5 years (Fig. 2b). The mean AKS functional score continuously improved until 2 years and was then constant until 5 years (Fig. 2c). The mean WOMAC pain score showed a significant improvement in pain until 1 year, a plateau until 2 years, then a marked decline between 2 and 5 years (Fig. 2d). The mean WOMAC stiffness score improved until 1 year then demonstrated a plateau phase until 5 years (Fig. 2e). The mean WOMAC function score showed improvement until 1 year and was then constant until 2 years, before declining between 2 and 5 years (Fig. 2f). The mean SF-36 physical component summary (PCS) score improved from baseline until 1 year and was constant until 2 years, before showing a decline at 5 years (Fig. 2g). The mean SF-36 mental component summary (MCS) score improved from baseline at 6 months, was constant until 2 years, then showed a marked decline between 2 and 5 years (Fig. 2h).

**Table 3:** Temporal patterns of functional outcome scales during the 5-year period

Score	Preop.	*p*-Value	PO 6 months	*p*-Value	PO 1 year	*p*-Value	PO 2 years	*p*-Value	PO 5 years
ROM	127.2 (1.4)	*< 0.001*	133.8 (0.7)	0.436	134.2 (0.8)	*0.043*	133.2 (1.0)	*< 0.001*	130.2 (1.0)
AKS									
Knee score (100)	44.4 (0.7)	*< 0.001*	95.2 (0.5)	*0.001*	93.5 (0.5)	*0.001*	95.0 (0.5)	*< 0.001*	92.5 (0.6)
Function score (100)	58.7 (1.0)	*< 0.001*	91.3 (0.9)	*< 0.001*	96.3 (0.8)	*0.040*	97.7 (0.6)	0.186	96.4 (0.9)
WOMAC									
Pain (20)	11.4 (0.3)	*< 0.001*	3.0 (0.3)	*0.012*	2.2 (0.2)	0.329	1.9 (0.3)	*0.002*	3.2 (0.4)
Stiffness (8)	4.8 (0.2)	*< 0.001*	2.3 (0.1)	*< 0.001*	1.7 (0.1)	0.192	1.5 (0.1)	0.166	1.8 (0.2)
Function (68)	39.5 (1.0)	*< 0.001*	18.7 (0.9)	*< 0.001*	14.4 (0.8)	0.242	13.5 (0.8)	*0.002*	17.7 (1.3)
SF-36									
PCS	29.3 (0.5)	*< 0.001*	41.3 (0.7)	*< 0.001*	45.4 (0.6)	0.356	44.6 (0.7)	*0.002*	42.0 (0.8)
MCS	42.0 (1.0)	*0.001*	46.8 (1.0)	0.086	48.9 (0.9)	0.854	49.1 (1.0)	*0.002*	44.8 (1.2)

*Preop.* preoperative, *PO* postoperative, *ROM* range of movement, *AKS* American Knee Society score, *WOMAC* Western Ontario and McMaster Universities Arthritis Index score, *SF-36* 36-Item Short Form Health Survey, *PCS* physical component summary, *MCS* mental component summary

Italics indicates significant *p*-values

Most of the functional outcome scores declined between 2 and 5 years in patients aged ≥ 68 years with an MB implant, after a variable initial improvement for 6 months to 2 years, whereas most of the parameters plateaued after 2 years in those aged < 68 years and in the older patients with an FB implant. In patients aged < 68 years with an FB implant, the AKS knee score, WOMAC pain score, WOMAC stiffness score, and WOMAC function score improved at 6 months and were constant thereafter until 5 years, whereas the AKS function score and SF-36 PCS showed improvement until 1 year before becoming constant up to 5 years (Table 4). The SF-36 MCS score tended to improve at 6 months (*p* = 0.062), after which it showed a constant value until 5 years. ROM did not show any improvement from baseline until 5 years. In the patients aged < 68 years with an MB implant, the ROM, AKS knee score, WOMAC stiffness score, and SF-36 MCS score improved at 6 months, whereas the AKS function score improved until 2 years and the WOMAC pain score, WOMAC function score, and SF-36 MCS showed improvement until 1 year. Except for ROM, which declined, and the WOMAC function score, which showed a tendency to decline (*p* = 0.092), all other scores were constant between 2 and 5 years (Table 5). In patients aged ≥ 68 years with an FB implant, ROM and AKS function scores improved at 6 months, whereas the WOMAC stiffness score improved until 1 year, and the AKS knee score, WOMAC pain score, WOMAC function score, and SF-36 PCS showed improvement until 2 years. The SF-36 MCS score, on the other hand, did not show any improvement from baseline until 5 years. The ROM and SF-36 PCS score declined, and the AKS knee score (*p* = 0.082) and WOMAC function score (*p* = 0.088) showed a tendency to decline between 2 and 5 years (Table 6). In patients aged ≥ 68 years with an MB implant, the ROM, AKS knee score, WOMAC pain score, and SF-36 MCS score improved at 6 months, whereas the AKS function score, WOMAC function score, and SF-36 PCS score improved until 1 year, and the WOMAC stiffness score improved until 2 years. All scores except the AKS function score declined between 2 and 5 years (Table 7).

**Table 4:** Temporal pattern of functional outcome scales in patients aged < 68 years with an FB implant

Score	Preop.	*p*-Value	PO 6 months	*p*-Value	PO 1 year	*p*-Value	PO 2 years	*p*-Value	PO 5 years
ROM	134.2 (2.1)	0.603	135.5 (1.9)	0.231	133.9 (2.5)	0.547	134.5 (2.8)	0.071	132.9 (2.5)
AKS									
Knee score (100)	48.1 (1.2)	*< 0.001*	95.8 (0.9)	0.209	94.6 (1.2)	0.615	95.1 (1.1)	0.069	92.1 (1.7)
Function score (100)	58.6 (2.2)	*< 0.001*	92.9 (1.7)	*< 0.001*	99.3 (0.5)	0.758	99.0 (0.9)	0.891	99.1 (0.9)
WOMAC									
Pain (20)	12.3 (0.7)	*< 0.001*	2.5 (0.5)	0.212	1.6 (0.5)	0.852	1.5 (0.4)	0.336	2.3 (0.7)
Stiffness (8)	4.8 (0.4)	*< 0.001*	2.0 (0.3)	0.122	1.4 (0.2)	0.653	1.6 (0.2)	0.916	1.6 (0.4)
Function (68)	39.6 (2.3)	*< 0.001*	13.4 (1.9)	0.342	11.9 (1.7)	0.780	11.5 (1.4)	0.673	12.6 (2.6)
SF-36									
PCS	31.2 (1.0)	*< 0.001*	44.6 (1.7)	*0.048*	48.1 (1.1)	0.436	46.8 (1.3)	0.272	44.5 (1.9)
MCS	39.8 (2.4)	0.062	45.7 (2.6)	0.484	48.2 (1.9)	0.798	49.0 (2.2)	0.152	44.1 (2.2)

*FB* fixed bearing, *Preop.* preoperative, *PO* postoperative, *ROM* range of movement, *AKS* American Knee Society score, *WOMAC* Western Ontario and McMaster Universities Arthritis Index score, *SF-36* 36-Item Short Form Health Survey, *PCS* physical component summary, *MCS* mental component summary

Italics indicates significant *p*-values

**Table 5:** Temporal pattern of functional outcome scales in patients aged < 68 years with an MB implant

Score	Preop.	*p*-Value	PO 6 months	*p*-Value	PO 1 year	*p*-Value	PO 2 years	*p*-Value	PO 5 years
ROM	127.0 (2.9)	*0.022*	133.3 (1.2)	0.198	134.4 (1.3)	*0.017*	132.2 (1.5)	*0.001*	128.3 (1.6)
AKS									
Knee score (100)	44.1 (1.9)	*< 0.001*	94.6 (1.2)	*0.003*	91.2 (1.2)	*0.006*	93.7 (0.9)	0.671	93.4 (1.1)
Function score (100)	59.7 (2.7)	*< 0.001*	95.0 (1.8)	0.722	95.6 (1.6)	*0.022*	98.1 (1.1)	0.521	98.1 (1.1)
WOMAC									
Pain (20)	10.6 (0.9)	*< 0.001*	3.9 (0.7)	*0.014*	2.4 (0.4)	0.806	2.3 (0.4)	0.718	2.1 (0.5)
Stiffness (8)	4.6 (0.4)	*< 0.001*	2.4 (0.3)	0.121	2.0 (0.3)	0.589	1.8 (0.2)	0.171	1.3 (0.2)
Function (68)	38.2 (2.6)	*< 0.001*	22.4 (2.0)	*< 0.001*	13.4 (1.2)	0.231	15.4 (1.4)	0.092	11.4 (2.1)
SF-36									
PCS	29.2 (1.3)	*< 0.001*	39.9 (1.6)	*0.001*	46.6 (1.2)	*0.023*	43.2 (1.3)	0.279	45.3 (1.2)
MCS	41.8 (1.5)	*0.002*	47.0 (1.9)	0.340	48.9 (1.5)	0.314	46.8 (1.8)	0.193	50.3 (1.8)

*MB* mobile bearing, *Preop.* preoperative, *PO* postoperative, *ROM* range of movement, *AKS* American Knee Society score, *WOMAC* Western Ontario and McMaster Universities Arthritis Index score, *SF-36* 36-Item Short Form Health Survey, *PCS* physical component summary, *MCS* mental component summary

Italics indicates significant *p*-values

**Table 6:** Temporal pattern of functional outcome scales in patients aged ≥ 68 years with an FB implant

Score	Preop.	*p*-Value	PO 6 months	*p*-Value	PO 1 year	*p*-Value	PO 2 years	*p*-Value	PO 5 years
ROM	122.6 (2.6)	*0.001*	133.0 (1.3)	0.313	134.0 (1.3)	*0.022*	132.0 (1.3)	*< 0.001*	127.5 (1.8)
AKS									
Knee score (100)	43.6 (1.2)	*< 0.001*	94.7 (0.9)	0.708	94.3 (0.8)	*0.010*	96.7 (0.6)	0.082	94.9 (0.8)
Function score (100)	59.2 (1.7)	*< 0.001*	88.7 (1.7)	0.173	91.6 (2.1)	0.517	93.0 (1.6)	0.367	95.3 (1.7)
WOMAC									
Pain (20)	11.2 (0.5)	*< 0.001*	2.9 (0.5)	0.420	3.3 (0.5)	*0.015*	2.0 (0.5)	0.177	3.4 (0.8)
Stiffness (8)	4.8 (0.3)	*< 0.001*	2.4 (0.2)	*0.014*	1.6 (0.2)	0.842	1.6 (0.2)	0.484	1.9 (0.4)
Function (68)	40.6 (1.8)	*< 0.001*	19.9 (1.6)	0.968	19.9 (1.7)	*0.013*	16.2 (1.5)	0.088	21.3 (2.9)
SF-36									
PCS	27.5 (0.7)	*< 0.001*	39.2 (1.2)	*0.043*	41.8 (1.1)	*0.027*	45.1 (1.4)	*0.001*	36.5 (2.2)
MCS	45.5 (1.9)	0.652	46.5 (1.9)	0.855	46.9 (1.9)	0.874	47.1 (2.0)	0.171	43.2 (2.6)

*FB* fixed bearing, *Preop.* preoperative, *PO* postoperative, *ROM* range of movement, *AKS* American Knee Society score, *WOMAC* Western Ontario and McMaster Universities Arthritis Index score, *SF-36* 36-Item Short Form Health Survey, *PCS* physical component summary, *MCS* mental component summary

Italics indicates significant *p*-values

**Table 7:** Temporal pattern of functional outcome scales in patients aged ≥ 68 years with an MB implant

Score	Preop.	*p*-Value	PO 6 months	*p*-Value	PO 1 year	*p*-Value	PO 2 years	*p*-Value	PO 5 years
ROM	126.2 (2.8)	*0.003*	133.5	0.362	134.3 (1.6)	0.900	134.2 (1.9)	*0.027*	132.0 (2.2)
AKS									
Knee score (100)	42.5 (1.3)	*< 0.001*	95.8 (1.1)	0.052	93.9 (1.0)	0.852	94.0 (1.1)	*< 0.001*	90.1 (1.2)
Function score (100)	57.6 (2.4)	*< 0.001*	89.5 (2.0)	*< 0.001*	98.9 (0.8)	0.944	98.8 (0.8)	0.837	95.2 (2.4)
WOMAC									
Pain (20)	11.4 (0.6)	*< 0.001*	2.6 (0.5)	0.069	1.5 (0.3)	0.297	2.2 (0.6)	*< 0.001*	4.3 (0.8)
Stiffness (8)	5.0 (0.3)	*< 0.001*	2.4 (0.2)	*0.013*	1.8 (0.2)	*0.035*	1.2 (0.2)	*0.001*	2.3 (0.3)
Function (68)	39.1 (1.6)	*< 0.001*	18.6 (1.3)	*< 0.001*	12.4 (1.3)	0.612	11.6 (1.4)	*< 0.001*	21.0 (2.0)
SF-36									
PCS	29.5 (0.8)	*< 0.001*	42.0 (1.4)	*0.013*	45.8 (1.4)	0.442	44.7 (1.3)	*0.016*	40.7 (1.2)
MCS	40.6 (1.8)	*0.003*	47.7 (1.9)	0.126	51.4 (1.6)	0.427	52.6 (1.5)	*< 0.001*	43.4 (2.0)

*MB* mobile bearing, *Preop.* preoperative, *PO* postoperative, *ROM* range of movement, *AKS* American Knee Society score, *WOMAC* Western Ontario and McMaster Universities Arthritis Index score, *SF-36* 36-Item Short Form Health Survey, *PCS* physical component summary, *MCS* mental component summary

Italics indicates significant *p*-values

## Discussion

Literature provides contradicting information regarding the temporal pattern of improvement in functional outcome after TKA. This uncertainty is further compounded by the fact that different outcome measurement tools are known to produce different results and have different capabilities [20]. Using generic, disease-specific, and performance-based measurements is therefore recommended to fully appreciate recovery after knee replacement [20, 21]. We prospectively evaluated functional outcome after uncomplicated TKA at various time points over 5 years using three different outcome measures, encompassing clinician- and patient-reported tools, besides ROM. We also identified differences, if any, in functional outcome after TKA over the same time points between FB and MB prostheses, and between patients aged ≥ 68 years as compared with their younger counterparts.

We found that most of the functional outcome parameters improved for 6 months to 2 years, followed by a downward trend between 2 and 5 years after uncomplicated TKA. Some previous studies have suggested that pain and function continue to improve for 4–7 years after TKA [5, 12]. A prospective study evaluating 49 total hip arthroplasty (THA)/TKA patients found a significant improvement in the WOMAC score and some components of the SF-36 score between 6 months and 7 years [5]. However, those authors did not study the outcome parameters between 6 months and 7 years, and this large gap in the timeline may mask important trends in the results, which could have important clinical implications. In our study, we evaluated the parameters at relatively short time intervals to address this limitation. Another study, evaluating perceived physical functioning in 44 THA/TKA patients by using the Hip Disability and Osteoarthritis Outcome Score (HOOS) and the Knee Injury and Osteoarthritis Outcome Score (KOOS) questionnaires, found a more significant improvement at 4 years than at 6 months [12]. However, no objective and clinician-reported outcome measures were used in that study. Moreover, the sample size was too small to make any definite conclusions, and the outcome was evaluated at fewer time points. We assessed functional outcome by using various outcome measurement tools, encompassing clinician- and patient-reported scores.

A few studies in literature suggest that improvements in functional parameters are limited to 1–2 years after TKA, after which they begin to decline [3, 8]. A prospective study, evaluating health-related quality of life (HRQOL) in 102 consecutive TKA patients by using the KOOS and SF-36 scores, found that the maximum improvement was evident at 1 year, after which the scores declined until 5 years [8]. However, that was a questionnaire-based study and did not consider objective clinical parameters. In our study, which assessed functional outcome using objective parameters, we also observed a decline in most scores between 2 and 5 years. Another study, evaluating functional results in 50 TKA patients by using KOOS and ROM measurements, reported that most of the improvements in pain and function were achieved by 6 months, with further small improvements up to 2 years before a small decline at 4 years [3]. Our findings closely resemble those of previous studies, while assessing function more elaborately and incorporating more patients. Although painful patellar clunk or crepitation (PCC) has been reported as one of the causes for late-onset pain after TKA, especially with contemporary posterior-stabilized prosthesis designs, no patients with an e.motion implant and only one patient with a Genesis II were diagnosed with PCC in a retrospective study of 948 primary posterior-stabilized TKAs [22]. Furthermore, the implant design of the two prostheses, including the shapes of the patellar component and trochlear groove, have not been specifically reported to be linked with knee pain or decreased ROM after TKA, thus ruling out implant-related bias in the decline of functional scores observed in this study.

We observed that the temporal pattern of functional outcome after TKA differed according to both implant type and patient age. Previously published studies on the effect of implant type (FB or MB) on the evolution of functional outcome after TKA could not find any such differences between the two implant groups [14, 23]. The theoretical advantage of MB TKA designs, due to increased congruity and unconstrained mobility of the bearing, has not been translated into improved long-term clinical outcomes or implant survivorship. A meta-analysis of the two TKA designs did not find any significant difference in clinical or radiological outcomes and complication rates between fixed- and mobile-bearing TKAs [14]. However, the above-mentioned study did not consider various time points in the recovery after TKA, which has important clinical implications. In our study, we evaluated the functional outcome after TKA in the two groups at various time points, to comprehensively evaluate patterns of recovery. A randomized controlled clinical trial comparing the outcomes of FB and MB TKAs found that most of the gait parameters improved at 5 years after TKAs using FB implants in older patients (> 70 years) and for TKAs using MB implants in younger patients [15]. However, their sample size was relatively small; moreover, they did not comprehensively study the temporal pattern of recovery in the various subgroups. We used MMRM to study the pattern of recovery in functional outcome at various time points, which has been reported to be a superior approach for controlling type I error rates and minimizing biases [24]. MB TKA designs have evolved over time, based on the range of freedom of the polyethylene insert, from the initial meniscal design to the rotating platform type, finally leading to the development of the anteroposterior glide platform variety. However, in a retrospective comparative study of the three types of mobile bearings, no difference in clinical or radiological results or implant survivorship was reported [25].

Only limited information is available in literature regarding comparison of the temporal pattern of functional outcome after TKA in older versus young patients. A prospective study comparing functional outcome and pain after TKA between patients aged ≤ 55 years and > 55 years at time of index surgery found no significant difference in pain or knee performance over 10 years of follow-up between the two groups. The authors, however, found higher overall function in the younger age group throughout the study period [26]. However, they did not evaluate the temporal pattern of recovery in the two groups at various time points. In our study, which had a relatively larger sample size, we found a decline in function between 2 and 5 years in the older patients (≥ 68 years) when implanted with an MB prosthesis. Considering the greater degree of freedom of movement provided by an MB implant compared with a fixed-bearing one, full function of the former implant appears to be more dependent on adequate quadriceps muscle power. The decline in functional outcome seen in older patients with an MB implant may be explained by decreased muscle strength associated with aging in such patients.

This study has a number of strengths, including use of a standardized clinical pathway, use of validated outcome measures, and 5-year follow-up. However, there are some limitations to this study, which should be considered when interpreting the results. First, we did not have a control group with which to compare the changes in functional outcome over the 5-year period. However, in an extensive literature review, we could not find any long-term prospective study that evaluated such outcome measures in a healthy population. Therefore, the above results can be assumed to be relevant to the general population. Second, as we only included unilateral TKAs in our study, effects of OA of the other knee on the serial outcome measures cannot be ruled out. However, to reduce this ambiguity, we excluded patients with severe OA of the other knee from analysis in the study. Moreover, this study provides empirical data that lay the path for future studies on bilateral TKA. Third, the lack of randomization regarding the type of prosthesis (FB or MB) used precludes the abolition of an inherent selection bias in the study. This should be borne in mind when interpreting the results. The use of a single type of FB or MB implant for all patients is another limitation that requires consideration, as this could be a potential source of selection bias.

In conclusion, a decline in function and pain relief occurs after 2 years following TKA. This decline is more evident in older patients (≥ 68 years) and in those with a MB prosthesis. Patients should be counseled preoperatively about the anticipated decline in function over time after 2 years following TKA. Based on these findings, we believe that use of MB implants in older patients (≥ 68 years) should be questioned. We further recommend future studies with larger sample size and longer follow-up to comprehensively evaluate the effect of MB implants in older patients.
